# Polymeric Dural Biomaterials in Spinal Surgery: A Review

**DOI:** 10.3390/gels10090579

**Published:** 2024-09-06

**Authors:** Taoxu Yan, Junyao Cheng, Qing He, Yifan Wang, Chuyue Zhang, Da Huang, Jianheng Liu, Zheng Wang

**Affiliations:** 1 Department of Orthopedics, Chinese PLA General Hospital, Beijing 100853, China; yantaoxu0409@163.com (T.Y.); cjyspine@163.com (J.C.); wyfspine@163.com (Y.W.); cycycyzhang301@163.com (C.Z.); 2 College of Biological Science and Engineering, Fuzhou University, Fuzhou 350108, China; 230820068@fzu.edu.cn (Q.H.); huangda@fzu.edu.cn (D.H.)

**Keywords:** biomaterials, low back pain, dural mater, anti-adhesion, tissue engineering

## Abstract

Laminectomy is a commonly performed surgical procedure by orthopedic and neurosurgeons, aimed at alleviating nerve compression and reducing pain. However, in some cases, excessive proliferation of fibrous scar tissue in the epidural space post-surgery can lead to persistent and intractable lower back pain, a condition known as Failed Back Surgery Syndrome (FBSS). The persistent fibrous tissue causes both physical and emotional distress for patients and also makes follow-up surgeries more challenging due to reduced visibility and greater technical difficulty. It has been established that the application of biomaterials to prevent epidural fibrosis post-lumbar surgery is more beneficial than revision surgeries to relieve dural fibrosis. Hydrogel-based biomaterials, with their excellent biocompatibility, degradability, and injectability and tunable mechanical properties, have been increasingly introduced by clinicians and researchers. This paper, building on the foundation of epidural fibrosis, primarily discusses the strategies for the preparation of natural and polymeric biomaterials to prevent epidural fibrosis, their physicochemical properties, and their ability to mitigate the excessive proliferation of fibroblasts. It also emphasizes the challenges that need to be addressed to translate laboratory research into clinical practice and the latest advancements in this field.

## 1. Introduction

Low back pain, a pervasive health issue transcending nationality and age, significantly contributes to global economic slowdown [1]. It is estimated that one in four adults experience transient low back pain [2], with approximately 600 million individuals worldwide suffering from this condition [3,4]. Among these patients, roughly 35% do not find relief from conservative, non-surgical treatments and require surgery to alleviate nerve compression caused by herniated disks or spinal stenosis [5,6].

Laminectomy, a common spinal surgical procedure, is one of the most effective methods for relieving neurogenic compression and associated lumbodorsalgia [7,8]. Failed Back Surgery Syndrome (FBSS), characterized by persistent and intractable pain in the lower back and extremities, affects about 5–10% of patients postoperatively [9,10]. FBSS is multifactorial, with epidural fibrosis being the primary etiology. The continuous traction on the unprotected dura by newly formed fibrotic tissue not only inflicts irreversible pain on the patient, but also complicates revision surgeries, presenting the operating surgeon with a more challenging field of view and increased procedural difficulty [11]. In surgeries for ossification of the ligamentum flavum, dural adhesion has also been confirmed as one of the significant causes of cerebrospinal fluid leakage and dural rupture. Consequently, the benefits of preventing epidural scar adhesions post-laminectomy are markedly superior to those of surgical adhesiolysis for postoperative adhesions.

In this comprehensive review, we have delineated the pathophysiological mechanisms underlying epidural fibrosis and the methodologies for adhesion assessment. The focus is on the development strategies, physicochemical properties, and clinical and fundamental applications of epidural anti-adhesion biomaterials. Additionally, we have discussed the advancements in their practical efficacy and other concomitant strategies.

## 2. Mechanics and Evolution of Dural Adhesion

Post-laminectomy epidural fibrosis is a normal physiological phenomenon of tissue healing. However, the extent of fibrosis can be mitigated by minimizing the length of the surgical incision, which is one of the most common causes of postoperative epidural fibrosis. The spinal cord in healthy individuals moves congruently with the direction of spinal motion without pain. In contrast, for patients with epidural fibrosis, the scar tissue can cause persistent pain, exacerbated by daily activities that increase traction on the dura mater and nerve roots, leading to chronic inflammation and intensified pain [12,13,14].

Tissue scar adhesion following laminectomy may be associated with inflammatory conditions, potentially mediated by an increased affinity of cells for collagen types I and IV, due to elevated levels of integrin subtype α2β1, and environmental factors such as Matrix Metalloproteinase-1 (MMP-1), Matrix Metalloproteinase-3 (MMP-3), and Vascular Endothelial Growth Factor (VEGF), and a decrease in Matrix Metalloproteinase-9 (MMP-9) [15,16].

In contemporary clinical practice, a myriad of strategies are employed to avert the occurrence of epidural scar adhesions. These encompass the refinement of surgical techniques, minimization of surgical incisions [17], utilization of both natural and synthetic hydrogels, application of electrospun films, implementation of artificial vertebral reconstruction procedures, epidural fat reimplantation, and pharmacological interventions. The latter includes the use of agents such as decorin (DCN) [18], hydroxycamptothecin [19,20], mitomycin-C [21,22], pirfenidone [23], and a spectrum of non-steroidal anti-inflammatory drugs exemplified by Ibuprofen [24].

Assessing the degree of adhesion is among the most critical steps in the experimental process. Three primary methodologies are typically employed: gross visual inspection, histological staining analysis, and magnetic resonance imaging (MRI). Gross visual examination utilizes a grading system to evaluate the relationship between the dura mater and surrounding tissues. Histological techniques such as H&E staining and Masson’s trichrome staining vividly delineate the morphological structure of cells and tissues, highlighting connective tissues and the degree of staining. MRI has become one of the pivotal tools for assessing epidural fibrosis, offering detailed insights into the structural composition of the epidural tissues [10,25,26].

## 3. Natural-Hydrogel-Based Biomaterials

Natural hydrogels, predominantly composed of renewable high-molecular-weight polymers from the natural world, are structured into three-dimensional networks, predominantly derived from polysaccharides and proteins. They exhibit exceptional biocompatibility and high water absorption, which has led to their remarkable performance across various sectors, including biomedical applications, cosmetics, and the agricultural industry. However, the adhesive and mechanical strength of natural hydrogels on moist surfaces, such as the dura mater, are insufficient, which significantly limits their in vivo applications. Consequently, the crosslinking of various natural materials and their chemical modification to enhance material properties have garnered widespread recognition among the scientific community [27,28,29].

### 3.1. Hyaluronic Acid

**Figure 1:**
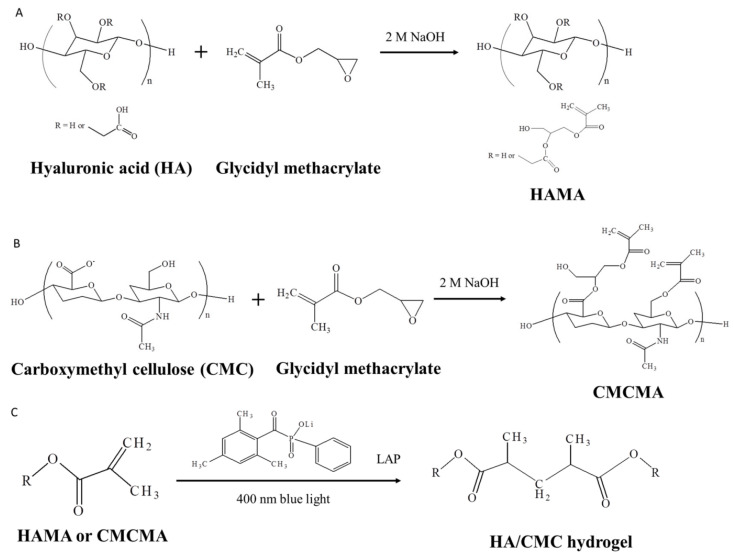
Application of natural hydrogels in dural tissue engineering. Synthesis of HAMA (**A**) and carboxymethyl cellulose methacrylate (CMCMA) (**B**) by reacting HA and CMC with glycidyl methacrylate. (**C**) Synthesis of HA/CMC hydrogel by photo-crosslinking reactions [36].

Hyaluronic acid (HA) hydrogels are a prevalent class of polysaccharide-based hydrogels, renowned for their natural origin. HA is ubiquitously distributed throughout the human body and has been utilized in biomaterials for nearly half a century [30,31]. These derivatives hold significant roles in a myriad of applications, including cellular delivery, molecular transportation, systemic targeted drug delivery, hemostasis and tissue repair, the regeneration of bone defects, and ophthalmic treatments [32,33,34,35]. Crosslinked hyaluronan, an absorbable adhesive barrier, has been extensively utilized in the postoperative prevention of tissue adhesion. Huang et al. synthesized a hydrogel by reacting HA and carboxymethylcelluose (CMC) with glycidyl methacrylate to form hyaluronic acid methacrylate (HAMA) and carboxymethyl cellulose methacrylate (CMCMA), respectively. The hydrogel, designated as HA/CMC (HC), was subsequently photo-crosslinked under 400 nm blue light. This HC hydrogel significantly extends the in vivo degradation time of HA hydrogels and allows for tunable degradation rates and extents by modulating the ratios of HA to CMC (Figure 1). Additionally, the hydrogel exhibited a marked reduction in both cellular adhesion and the in vitro infiltration of fibroblasts, which are pivotal in the formation of adhesions, thereby underscoring its prospective anti-adhesive capabilities within a biological milieu. Within a four-week period, the hydrogel exhibited superior efficacy in preventing epidural scar adhesion in a New Zealand white rabbit model and may serve as a promising material for dural repair [36].

Researchers, including Ji et al., synthesized an injectable hydrogel using pyridinone (PFD) and HAMA at an optimal concentration. This hydrogel is capable of the sustained release of PFD, which exerts an anti-fibrotic effect by inhibiting the formation of collagen both in vitro and in vivo. The study demonstrates that PFD-loaded HAMA hydrogel effectively suppresses the infiltration of cells such as fibroblasts, macrophages, and neutrophils, and mitigates the generation of reactive oxygen species (ROS) and inflammatory mediators within the body. PFD-loaded HAMA hydrogel has been proven to be safe and efficacious, exhibiting remarkable anti-adhesion properties in a rat model where the L1-L2 laminae were resected. Additionally, this hydrogel exhibits an appropriate degradation rate, a crucial characteristic for a successful anti-adhesion hydrogel. The mass of the hydrogel decreased from an average of 0.81 g pre-implantation to an average of 0.25 g after 8 weeks [23]. In preliminary experiments, pirfenidone has been shown to mitigate fibroblast proliferation, migration, and adhesion through the inhibition of the PI3K/AKT signaling pathway. This pathway is integral to cellular processes such as survival, metabolism, proliferation, migration, adhesion, and protein synthesis. An elevated concentration of pirfenidone (PFD) markedly suppresses fibroblast proliferation. PFD may represent a safe and efficacious candidate for the reduction in clinical epidural fibrosis and holds promise for repurposing in various clinical applications [37].

Lin et al. developed an ibuprofen-conjugated HA–PGA hydrogel capable of in situ crosslinking for localized drug delivery. This HA–PGA hydrogel, conjugated with ibuprofen, demonstrated favorable biocompatibility and efficacy in reducing lipopolysaccharide-induced prostaglandin E2 production. The utilization of ibuprofen within the hydrogel serves to delay the coagulation of the dura mater and mitigates the proliferation of giant cells and collagen fibers [38].

### 3.2. Chitosan

**Figure 2:**
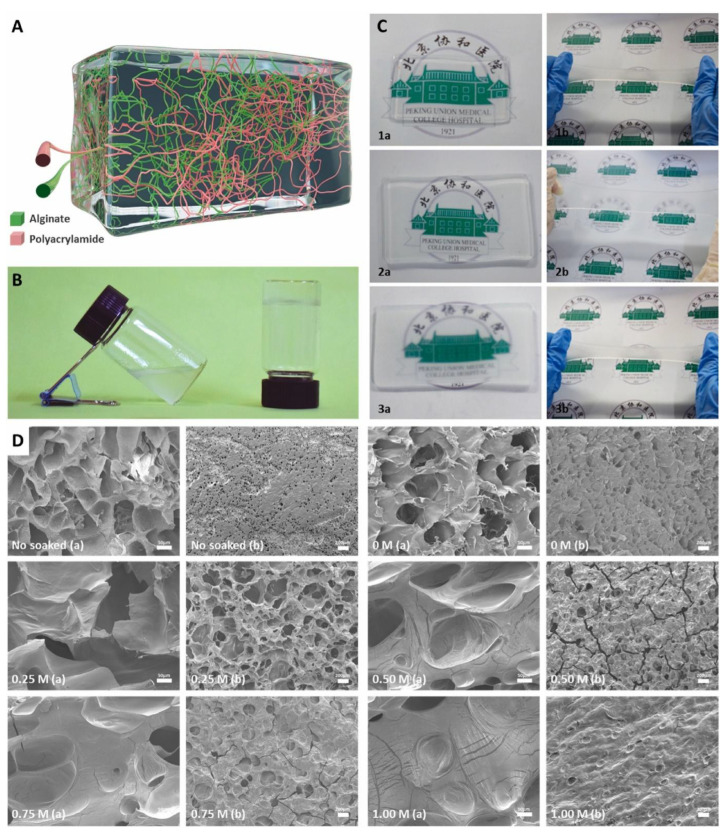
Application of natural hydrogels in dural tissue engineering. Macroscopic and microscopic structure of patch matrix. (**A**) Schematic diagram of cross-linking structure of patch matrix. Green: sodium alginate. Red: polyacrylamide. (**B**) Images of patch matrix before (left) and after (right) coagulation. (**C**) Photographs of patch matrix standing and stretching. 1: Patch matrix just removed from mold. 2: Patch matrix soaked in water for 24 h. 3. Patch matrix soaked in 0.25 M calcium chloride for 24 h. a: standing. b: stretched. (**D**) SEM photos of patch matrix soaked in different concentrations of calcium chloride. No soaked: patch matrix just removed from the mold. 0/0.25/0.50/0.75/1.00 M: patch matrix soaked in 0/0.25/0.50/0.75/1.00 M calcium chloride for 24 h. a: cross section. b: surface. Reproduced with permission from ELSEVIER, Copyright 2022 [43].

Chitosan-derived materials, endowed with optimal degradability, superior biocompatibility, and potent antimicrobial attributes, are deemed an ideal selection of natural biomaterials for the prophylaxis of FBSS [39,40,41]. A novel thermosensitive anti-adhesive gel, composed of a physical blend of locust bean gum, chitosan, and gelatin, has been shown to exhibit comparable efficacy in preventing adhesion to that of HA-based pharmaceuticals [42]. Li et al. presents an anti-inflammatory, antibacterial, and analgesic bioactive patch composed of a calcium ion-crosslinked alginate and polyacrylamide hydrogel matrix, along with a chitosan adhesive (Figure 2). This patch achieves a seal of the dura mater through the application of minimal pressure. In subsequent in vivo experiments, the adhesive also diminishes the expression of Glial Fibrillary Acidic Protein (GFAP), Ionized Calcium Binding Adapter molecule-1 (IBA-1), Myelin Basic Protein (MBP), Tumor Necrosis Factor-alpha (TNF-α), and Cyclooxygenase-2 (COX-2), thereby serving to alleviate pain, reduce inflammation, and prevent adhesion of the dura mater [43].

In a study conducted by Vediappan et al., chitosan was selected as a carrier for the iron chelator deferiprone (Def). Def, commonly utilized in the treatment of hematological disorders, has been demonstrated to diminish both inflammatory cells and reactive oxygen species (ROS). A linear correlation was observed between the proliferation of fibroblasts in vitro and the duration and concentration of DEF exposure. During surgery, the L1-L5 laminar segments of male Merino sheep were excised, exposing the dorsal surface of the dura mater to an approximate area of 2 cm by 1 cm and treated with 0.5 g of Kaolin. Animals were randomly divided into five groups, with each laminectomy level receiving one of the following treatments: no treatment, chitosan, chitosan with 20 mM Def, chitosan with 40 mM Def, or a carboxymethylcellulose and polyethylene oxide (CMC/PEO) hydrogel. The results indicated that Merino sheep treated with chitosan containing 20 mM Def exhibited the most effective prevention of dural adhesion, underscoring the potential application of chitosan combined with Def in the prevention of epidural scar adhesion and the avoidance of FBSS [44].

### 3.3. Collagen

Collagen, a family of high-strength, naturally occurring proteins, is ubiquitously present in the natural world and plays an integral role in the human body due to its exceptional tensile properties. It is instrumental in maintaining the mechanical integrity of various tissues and constitutes a significant component of the extracellular matrix in ligaments, skin, osseous tissue, and other connective tissues [45,46,47,48].

**Figure 3:**
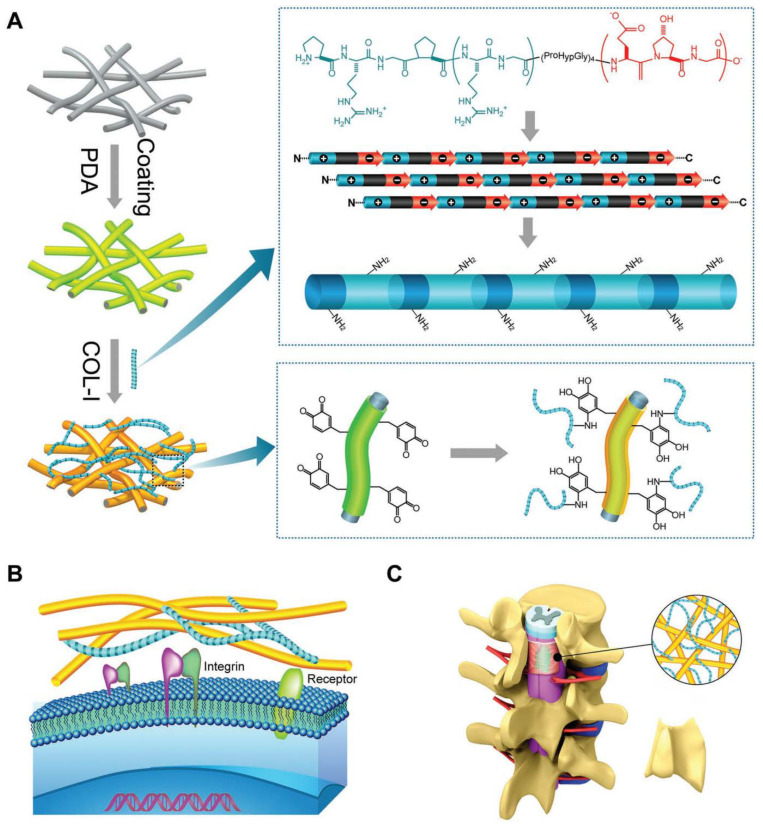
Application of natural hydrogels in dural tissue engineering. Schematic illustration of fabricated process of hierarchical micro/nanofibrous scaffolds, and using scaffolds with special function to repair dural defect and prevent epidural scarring. (**A**) Electrospun microfibrous matrices coated with mussel-inspired polydopamine, and process of collagen self-assembly to form nanofibers. (**B**) Modulation of cell behavior and activating functional response of cells and tissues. (**C**) In vivo spinal dural defect model is used to demonstrate that designed biomimetic scaffolds would promote generation of dura mater and prevent epidural scarring. Reproduced with permission from Wiley, Copyright 2017 [49].

Xu et al. fabricated a novel biomimetic fibrous scaffold with a stable stratified structure through the self-assembly of type I collagen molecules, coupled with electrospinning and polydopamine (PDA) coating crosslinking strategies (Figure 3). This scaffold integrates dual-component fibers at both micro- and nanoscales, emulating the heterogeneous micro/nano-architecture of the dura mater. The resultant bio-scaffold is conducive to the regeneration of large, continuous, and naturally analogous dura mater tissues, thereby facilitating the repair of dural defects. Magnetic resonance imaging (MRI) at 8 weeks postoperatively revealed continuous linear dura mater in the SF-PDA-COL scaffold group, with no evidence of dural adhesion observed in the sagittal MRI. Furthermore, MRI at 24 weeks demonstrated a distinct gap between the dura mater and surrounding musculature. Comparatively, the SF-PDA-COL scaffold group exhibited the lowest adhesion scores across all control groups. Collectively, these findings underscore hierarchical micro/nanofibrous scaffolds’ dual functionality as an effective dural substitute, with the added benefit of preventing postoperative epidural scar adhesion [49].

**Figure 4:**
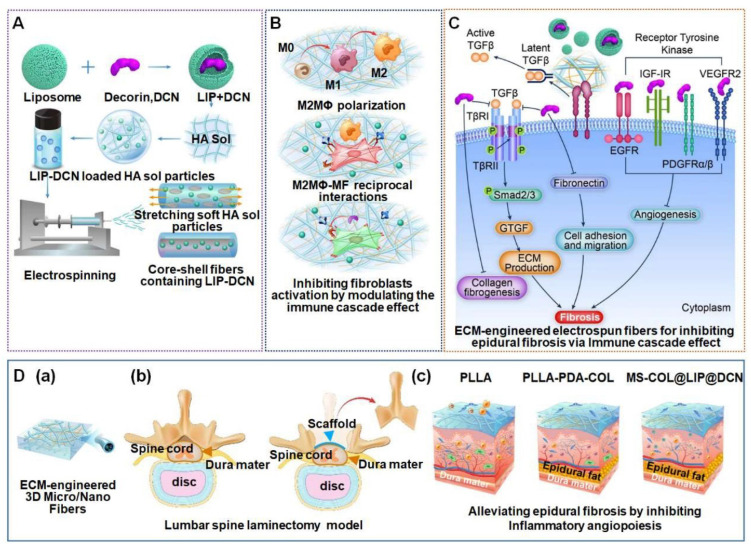
Application of natural hydrogels in dural tissue engineering. ECM-engineered micro/nanofibrous scaffold with immune cascade effect for inhibiting epidural fibrosis. (**A**) Construction of ECM-engineered scaffold using DCN-loaded liposome, microsol electrospinning, and self-assembly of type I collagen and its application in laminectomy surgery. (**B**) ECM-engineered scaffold regulating balanced M1/M2 phenotype activity through immune cascade effect. (**C**) Biological mechanism of ECM-engineered scaffold inhibiting epidural fibrosis. (**D**) ECM-engineered scaffold alleviating epidural fibrosis by inhibiting inflammatory angiogenesis. (a) ECM-engineered 3D micro/nanofibers. (b) Lumbar spine laminectomy model. (c) Alleviating epidural fibrosis by inhibiting inflammatory angiopoiesis and regulating immune cascade effects. Reproduced with permission from Elsevier, Copyright 2023 [51].

An ECM-engineered scaffold, meticulously constructed using DCN, micro/nanofibrous electrospun meshes, and self-assembled type I collagen, was designed to mitigate epidural fibrosis through the modulation of the immune cascade effect (Figure 4). This intricate system is adept at balancing the activation of M1 and M2 macrophages during the tissue repair process, thereby preventing excessive M2 activity that could lead to tissue fibrosis. Furthermore, DCN, a pivotal component of this scaffold, exerts its antifibrotic influence by antagonizing TGF-β1 through the TGF-β/Smad3 signaling pathway, thereby inhibiting the fibrotic activity of fibroblasts [50]. Transforming growth factor-beta 1 (TGF-β1) exerts a significant influence on the development of epidural scar adhesions through the stimulation of fibroblast proliferation, transdifferentiation into myofibroblasts, and an overabundance of extracellular matrix (ECM) protein deposition. DCN, a natural antagonist of TGF-β1, has been widely utilized for its anti-adhesive properties postoperatively across various medical fields. Researchers have successfully delineated the efficacy of DCN in a rat model following laminectomy. MRI assessments at 4 and 8 weeks post-treatment revealed that the DCN-administered group exhibited only a minimal amount of dense scar tissue around the dural sac, with no signs of compression. Histological analysis further corroborated these findings, with the DCN treatment group receiving the lowest score for epidural fibrosis, substantiating the significant inhibitory effect of DCN on the proliferation of epidural fibroblasts [51].

## 4. Synthetic Polymer-Based Biomaterials

The deployment of synthetic polymers within the medical sector has witnessed marked escalation, attributable to their malleable attributes that facilitate their adaptation to bespoke biomedical endeavors. These substances are frequently favored for their amenable interaction with biological systems, robust mechanical resilience, and plasticity in structural configuration, rendering them optimally suited for an array of medical interventions. Such applications encompass the realms of tissue engineering and pharmaceutical compound dispensation mechanisms, and they are also increasingly being utilized in advanced therapies and diagnostics [52,53,54].

### 4.1. PLGA

PLGA, polylactic-co-glycolic acid, a biodegradable polymer synthesized through the copolymerization of polylactic acid (PLA) and polyglycolic acid (PGA), is renowned for its exceptional biocompatibility, tunable degradation rate, and adjustable physicochemical properties. These characteristics have propelled its widespread application across the biomedical spectrum [55,56,57]. In the research undertaken by Fan and colleagues, PLGA-g-PVP(Polyvinylpyrrolidone)/PC(Phosphatidylcholine) nanofiber membranes were meticulously crafted via the electrospinning technique. This innovative process substantially augmented the hydrophilicity of the PLGA nanofibers (NFm), thereby efficaciously attenuating the environmental perturbations stemming from pH fluctuations induced by degradation byproducts. Moreover, the procedure markedly diminished the incidence of inflammation and tissue adhesion. In addition, the novel formulation Interleukin curtailed the mucosal irritation commonly linked with PVP/I, culminating in a more biocompatible material primed for medical utilization. PLGA-g-PVP/PC has exhibited remarkable anti-inflammatory and antibacterial attributes, achieving superior efficacy in the prevention of adhesions in the dura mater [58].

**Figure 5:**
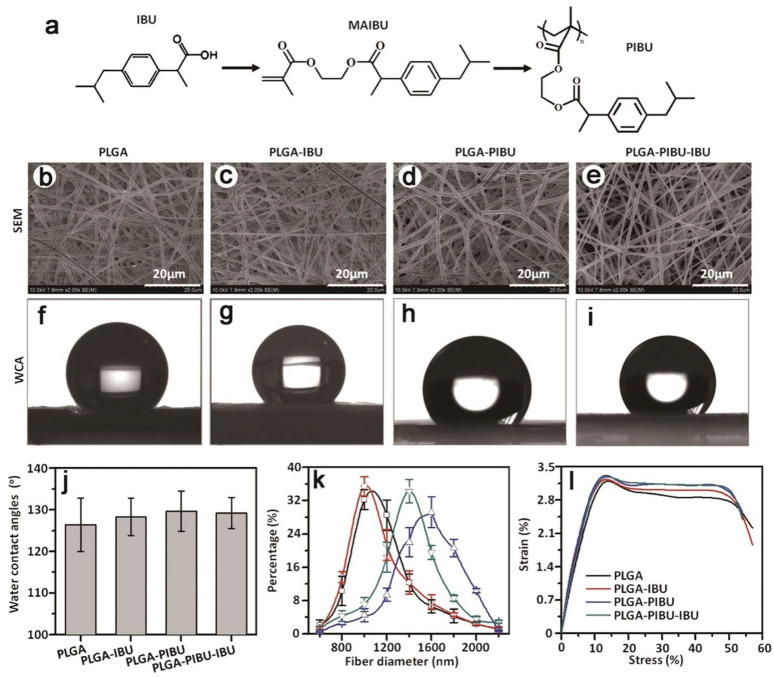
Application of synthetic hydrogels in dural tissue engineering. Preparation and characterization of different fibrous membranes. (**a**) Synthesis procedure of PIBU. (**b**–**e**) SEM morphology of PLGA, PLGA-IBU, PLGA-PIBU, and PLGA-PIBU-IBU fibers. (**f**–**i**) Water contact angle images of PLGA, PLGA-IBU, PLGA-PIBU, and PLGA-PIBU-IBU fibrous membranes. (**j**) Water contact angle values for PLGA-IBU, PLGA-PIBU, and PLGA-PIBU-IBU fibers (n = 3, mean ± SD). (**k**) Diameter distribution of PLGA, PLGA-IBU, PLGA-PIBU, and PLGA-PIBU-IBU fibers (n = 3, mean ± SD). (**l**) Representative mechanical (strain %) curves of PLGA, PLGA-IBU, PLGA-PIBU, and PLGA-PIBU-IBU fibrous membranes. Reproduced with permission from Elsevier, Copyright 2017 [24].

Researchers engineered a PLGA/CS scaffold via a chemical crosslinking methodology, which capitalizes on the electrostatic interactions of the amide bonds to enhance the scaffold’s stability. This approach effectively improved the hemostatic properties and significantly reduced the occurrence of epidural scar adhesions (Figure 5). The study’s outcomes suggest that the PLGA/CS scaffold is an efficacious option for the prevention of postoperative epidural scar adhesions [59]. Ibuprofen (IBU) and poly(hydroxyethyl methacrylate) (PHEMA) were synthesized to form an ibuprofen prodrug (PIUB) via the electrospinning technique, which was then integrated onto a PLGA substrate, yielding a novel PLGA fibrous physical film barrier. This construct sustains the release of IBU for approximately eight weeks in vitro, preventing epidural scar adhesion and inflammatory responses by inhibiting the COX-2 pathway [24].

In a recent study, Yue et al. synthesized PLA-MFQ grafted membranes through the technique of electrospinning, utilizing polylactic acid (PLA) grafted with mefloquine (MFQ). These membranes exhibit an initial burst release of mefloquine, followed by a sustained release, which serves to prevent postoperative adhesion. Furthermore, the polylactic acid–mefloquine grafted electrospun fibrous membranes demonstrated potent anti-inflammatory properties, significantly reducing the expression of inflammatory markers (IL-1β and TNF-α) in macrophages and neo-vasculature within the surgical site [60].

### 4.2. PCL

Polycaprolactone (PCL) is a biocompatible and biodegradable polymeric material that is distinguished by its inherently slow degradation kinetics within biological environments. This polymer is frequently selected for a myriad of medical applications, including, but not limited to, drug delivery systems, where its sustained release properties are advantageous. Additionally, PCL’s malleable nature allows for its use in the fabrication of scaffolds for tissue engineering, as well as in the development of orthopedic devices and sutures that demand a balance of strength and flexibility [61,62]. Andrychowski and colleagues reported on the fabrication of a nanofibrous mesh barrier composed of poly(L-lactide-co-caprolactone) (PLCL) via the electrospinning technique, which was demonstrated to mitigate excessive scarring within the surgical site post-laminectomy [63]. Shi et al. engineered a bilayered, drug-impregnated electrospun nanofiber membrane, tailored to prevent epidural scar adhesion. This composite was synthesized from a blend of polycaprolactone (PCL) and chitosan (CS), leveraging the precision of electrospinning technology. The construct boasts a meloxicam-enriched lower stratum, capitalizing on its potent anti-inflammatory properties, and an upper stratum suffused with mitomycin-C, designed to suppress DNA and collagen synthesis. Over the course of approximately 12 days, the stratified membrane ensured the efficacious release of both pharmaceutical agents. This synergistic strategy efficaciously forestalled the onset of epidural scar adhesions, concurrently mitigating inflammatory responses and attenuating the expression of collagen types I and III [64].

The icariin-loaded polycaprolactone (PCL) and gelatin fibers were fabricated into nanofibrous membranes via an innovative and highly efficient electrospinning technique. The amalgamation of PCL with natural polymers such as gelatin may yield superior ultrafine fibers, characterized by enhanced biodegradability and hydrophilicity. The ICA-loaded PCL–gelatin electrospun membrane serves as an effective anti-adhesion barrier through the controlled release of icariin, which modulates the transforming growth factor-beta and Smad signaling pathways to inhibit the proliferation of fibroblasts and downregulate the expression of collagen I/III and α-smooth muscle actin (α-SMA) [65].

### 4.3. PEG

Polyethylene glycol (PEG) is a versatile, water-soluble polymer that has found broad application within the medical and pharmaceutical industries, attributable to its biocompatibility, non-toxicity, and formulation adaptability [66,67,68,69]. Coseal, a commercially available hemostatic polyethylene glycol (PEG) hydrogel, is utilized as an adhesive in vascular reconstruction and is also recognized for its efficacy in preventing postoperative adhesion following cardiac and intra-abdominal surgeries. In an experimental application post-laminectomy, KESKİN et al. demonstrated that Coseal is effective in preventing epidural scarring adhesion. A histopathological examination of tissue sections confirmed the utility of Coseal in preventing adhesions in the epidural space [70]. Li et al. synthesized a thermogel polymer, PLGA-PEG-PLGA, to investigate its efficacy in preventing epidural scar adhesion. The PLGA-PEG-PLGA thermogel demonstrated utility in a rat model of dural adhesion, suggesting its potential as an effective protective material against postoperative adhesion following intra-abdominal surgery [71].

## 5. Other Strategies

Chen et al. engineered a composite sponge by integrating biodegradable CMC with Bletilla striata polysaccharide (BSP) and resveratrol (RES), culminating in a bilayer CMC sponge that exhibits potential for hemostasis, anti-fibrotic effects, and anti-adhesion properties. The sponge evinced remarkable biocompatibility and demonstrated an ability to downregulate the expression of S100A4 and P4HB in NIH/3T3 cells. This suggests that the CMC-BSP-RES sponge may effectively curtail fibroblast activity, thereby serving as a proficient preventive measure against adhesion [72].

**Figure 6:**
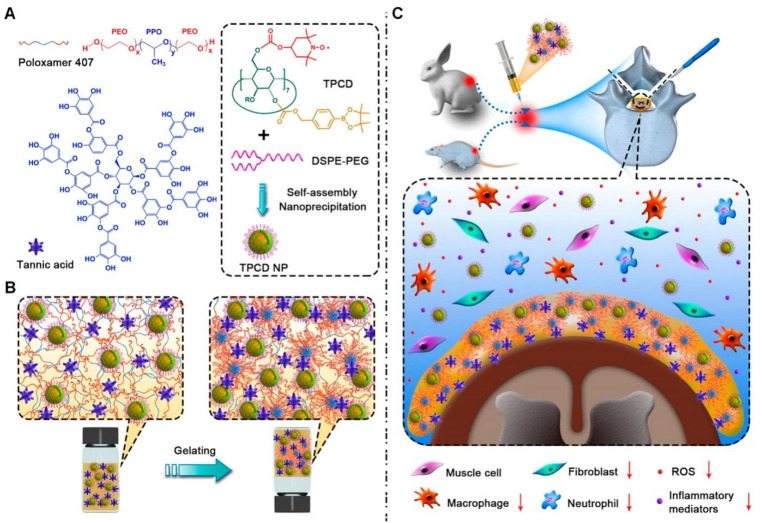
Schematic illustration of engineering of multifunctional hydrogel for prevention of postoperative epidural fibrosis after lumbar laminectomy. (**A**) Chemical structures of three functional components for construction of multifunctional hydrogels. (**B**) Sketch showing temperature-responsive sol−gel transition of newly designed hydrogel formulation. (**C**) Prevention of epidural fibrosis and adhesion post-laminectomy by local treatment with engineered advanced hydrogel. Reproduced with permission from American Chemical Society, Copyright 2020 [73].

Researchers have meticulously selected optimal concentrations of Poloxamer 407 (PX), a thermosensitive triblock copolymer; TPCD NP, a nanoparticle with reactive oxygen species (ROS) elimination and anti-inflammatory properties; and tannic acid (TA), a compound that enhances adhesion, to engineer an advanced injectable multifunctional supramolecular hydrogel denoted as PXNT (Figure 6). PX, utilized as the hydrogel-forming material, exhibits a pronounced thermosensitive sol–gel transition below 37 °C. The TPCD NP nanoparticles possess antioxidant attributes, effectively neutralizing ROS and mitigating oxidative stress, concurrently contributing to the hydrogel’s anti-inflammatory profile by diminishing the expression of inflammatory cytokines. The incorporation of TA significantly bolsters the hydrogel’s tissue adhesivity, ensuring secure retention at the surgical site and reducing the likelihood of displacement. The PXNT hydrogel demonstrates a remarkable capacity to attenuate local inflammatory responses and oxidative stress, endowed with multifaceted functionalities such as in situ thermosensitive gelation, self-healing, and bioadhesion. It has shown exceptional efficacy in preventing epidural fibrosis and adhesion post-laminectomy in both rabbit and rat models. Compared to other biomaterials developed for the prevention of post-laminectomy adhesion, the PXNT hydrogel offers broader coverage for surgical sites, superior adhesivity to prevent detachment during movement, an optimal residence time within the body, desirable viscoelasticity that facilitates spinal mobility without causing compression of the spinal cord, and excellent biocompatibility with no adverse effects or foreign body reactions. Furthermore, the PXNT hydrogel holds potential for preventing postoperative adhesions in various other surgical contexts, including cardiac, hepatic, splenic, and renal surgeries, as well as within the abdominal and uterine cavities [73].

**Figure 7:**
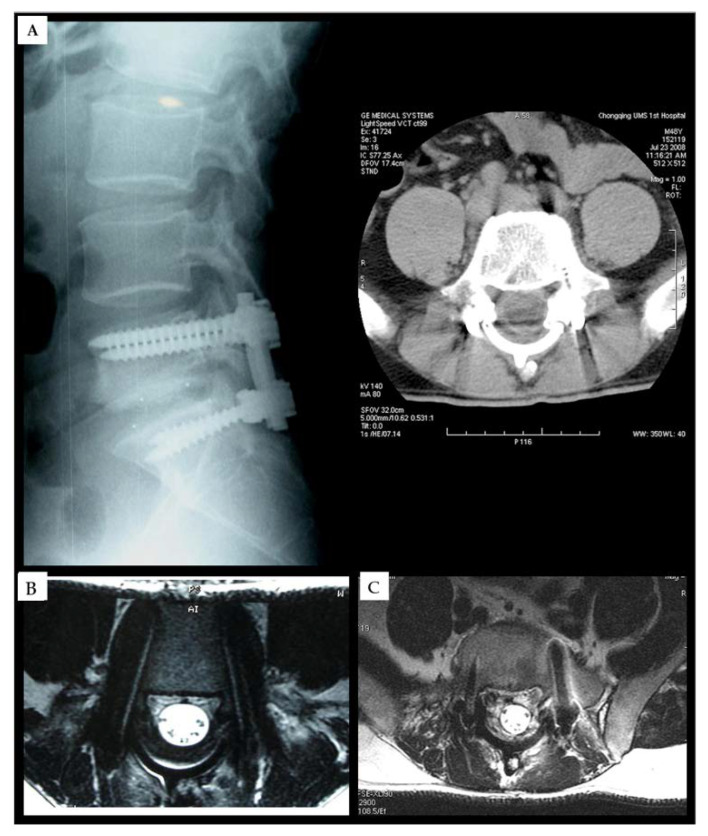
(**A**) The postoperative X-ray films showed that the internal fixation was in a good position with a good spinal alignment. (**B**) The postoperative CT and MRI of the post-op showed that the lumbar vertebral canal had good morphology and was covered by the n-HA/PA66 artificial lamina. (**C**) The postoperative axial MRI of the post-op and follow-up at 5 years showed no epidural scar formation and no compression of the nerve root. Reproduced with permission from Medical Science Monitor, Copyright 2018 [75].

In this study, hydroxyapatite (HA) laminae with distinct surface topographies were fabricated utilizing the cold isostatic pressing (CIP) and slip casting (SC) methodologies. The HA-CIP surface exhibited a higher degree of compaction compared to the HA-SC surface. Both biomaterials demonstrated a capacity to promote bone tissue regeneration and repair following laminectomy, facilitating the infiltration of proliferative fibrous tissue into the post-excision lamina region. Additionally, they effectively mitigated the occurrence of epidural fibrosis and scar adhesion (Figure 7). In a rabbit model, the HA-CIP displayed superior anti-adhesion properties compared to the HA-SC [74]. Artificial laminoplasty represents a promising approach to prevent postoperative epidural scar adhesion. Researchers have developed a composite material known as N-HA/PA66, which is synthesized from bioactive ceramics n-HA and organic polymer PA to emulate natural bone. This composite was utilized in the reconstruction of the spinal canal following laminectomy in patients with lumbar spinal stenosis and lumbar disk herniation, serving as a physical barrier to achieve anti-adhesion effects. It was approved for clinical use by the China National Medical Products Administration in 2005. A follow-up period of 4 to 7 years, with an average of 5.2 years, was conducted for the patients. Postoperative assessments were performed at 3-, 6-, and 12-month intervals using plain radiography, computed tomography (CT), or magnetic resonance imaging (MRI) to evaluate the lumbar region. The N-HA/PA66 composites were observed to be fully integrated and retained postoperatively. Radiographic findings indicated optimal positioning of all internal fixation devices, with no signs of screw loosening. MRI evaluations revealed complete expansion of the thecal sac, with no evidence of nerve root compression or epidural scarring. The application of N-HA/PA66 in post-lumbar surgery has demonstrated remarkable outcomes. This indicates that this novel biomaterial holds potential for translational value from the laboratory to clinical applications [75].

Derived from a patient’s own venous blood, Platelet-Rich Plasma (PRP) is an autologous concentrate replete with a plethora of growth factors, such as Platelet-Derived Growth Factor (PDGF), VEGF, Epidermal Growth Factor, Platelet Factor 4 (PF-4), Insulin-Like Growth Factor-1 (IGF-1), and Transforming Growth Factor-beta (TGF-β) [76]. These bioactive agents have been extensively utilized in spinal surgery applications, including lumbar disk herniation, spinal cord injury, spinal fusion, and the promotion of degeneration in the intervertebral disk. Despite the auspicious prospects of PRP across various medical domains, its efficacy remains inconsistent within specific areas. Notably, researchers have reported on the efficacy of PRP in preventing epidural scar adhesions post-laminectomy [77,78,79]. The study’s findings suggest that PRP possesses superior bio-physical barrier properties when compared to hyaluronic acid (HA) and activated Polyethylene Glycol (PEG) substitutes, thereby offering significant potential in the mitigation of epidural scar adhesions. These adhesions, which are characterized by the formation of scar tissue subsequent to spinal surgery, can lead to chronic pain and a cascade of complications. The pronounced efficacy of PRP in reducing the prevalence of such adhesions is predominantly attributed to its growth factors, which are instrumental in facilitating a scarless healing process. This unique characteristic positions PRP as an exceptionally beneficial agent in the postoperative care regimen following spinal surgeries, emphasizing its indispensable role in the maximization of favorable patient outcomes [80,81].

Loose adipose tissue can also effectively prevent epidural fibrosis induced by laminectomy, whether it is placed around the dura mater or around the nerve roots [14,82]. Lin et al. developed an injectable HA hydrogel, fortified with decellularized adipose matrix (DAM) and adipose-derived stromal cells (ASCs), with the objective of reconstructing epidural fat to prevent epidural fibrosis and adhesion. This composite hydrogel, integrating DAM and ASCs, is engineered to encompass all essential elements for the regeneration of epidural fat. Following implantation, the HA functions as a physical barrier to impede the ingress of proliferative fibrous tissue. Concurrently, the DAM initiates the differentiation of ASCs into adipocytes. As the HA hydrogel degrades over time, it is progressively replaced by the newly formed adipose tissue, which then serves as a barrier to prevent epidural scar adhesion. Although the DAM/ASC-enriched HA hydrogel demonstrated potential in reducing adhesion, further studies in larger animal models are imperative to substantiate its efficacy and safety [83].

## 6. Conclusions and Prospects

Epidural scar adhesion following laminectomy is one of the most significant contributors to FBSS, presenting a myriad of challenging issues for both patients and clinicians. In this comprehensive review, we distill insights from in vitro cellular assays, animal models post-laminectomy, and clinical observations to elucidate the practical efficacy and underlying mechanisms of existing materials designed to prevent epidural scar adhesions and mitigate excessive epidural fibrosis following laminectomy. In this review, we have delineated the pathophysiological mechanisms leading to FBSS following laminectomy, the signaling pathways and biological factors that influence epidural scar adhesion, and methodologies for adhesion assessment and grading. A focused discussion is presented on the practical applications of both natural and synthetic polymeric biomaterials in the context of post-laminectomy procedures. The current preventive strategies for FBSS encompass a spectrum of approaches, including refining surgical techniques, abbreviating operative duration, employing physical barriers, artificial vertebral reconstruction, drug delivery systems, and epidural fat reimplantation. The principal tactic in averting epidural fibrosis is to curtail fibroblast migration, ensure thorough hemostasis in the surgical field, and segregate the dura mater from fibrous tissues. While commercially available anti-adhesion materials have gained clinical approval, their high cost, suboptimal adhesion, and inadequate mechanical strength underscore an ongoing and pressing need in modern spinal surgery for innovative anti-adhesion materials to prevent postoperative epidural fibrosis.

The optimal epidural anti-adhesion material should exhibit highly tunable viscoelastic mechanical properties, including adjustable shear thinning capabilities and rapid self-healing, enabling straightforward application via standard devices such as simple spraying or spreading, or deployment through catheter delivery or direct injection. These materials also possess excellent tissue adhesion to ensure localized retention over clinically relevant timeframes and demonstrate high biocompatibility. Moreover, they maintain viscoelasticity, allowing organs and tissues to move freely relative to each other, effectively preventing adhesion.

The mechanical property enhancement of existing known materials has gained increasing acceptance among clinicians and researchers. However, the journey from the conception of a novel biomaterial to its clinical application is fraught with challenges. To date, no sufficiently outstanding and rational solutions have been presented to address this clinical conundrum. This article aims to provide a deeper understanding of the clinical intricacies associated with post-dural puncture adhesion prevention. It is anticipated that further investment in research and funding will be directed towards the field of dural adhesion prevention, ultimately benefiting a broad spectrum of patients. With ongoing research and technological advancements, the successful translation of preclinical studies into clinical practice is envisioned for the future.

## Data Availability

No new data were created or analyzed in this study. Data sharing is not applicable to this article.
